# The CLEAR model: a structured communication protocol for breast radiologists

**DOI:** 10.3389/frhs.2026.1890985

**Published:** 2026-08-27

**Authors:** Teresa Abbattista, Aram Hasan

**Affiliations:** 1 Breast Diagnostic Unit, “Principe di Piemonte” Hospital, AST Ancona, Senigallia, Italy; 2 United Arab Emirates University Department of Cognitive Sciences, Al Ain, United Arab Emirates

**Keywords:** breast, breast imaging, communication, method, training path

## Abstract

**Background:**

Communication in diagnostic breast imaging remains a critical yet frequently neglected aspect of radiological training. Traditionally centered on technical image interpretation, radiological education often leaves practitioners unprepared to navigate the severe emotional and psychological burden of a suspected breast cancer diagnosis.

**Objectives:**

This paper advocates for the communicative autonomy of the breast radiologist, arguing that direct, empathetic communication is not a secondary skill, but a core clinical act that directly influences both the psychological wellbeing and the physiological stress response of the patient.

**Methods:**

We propose the **CLEAR protocol** (*Connect, Listen, Explain, Ask, Reassure*), a transcultural framework conceptualized to bridge clinical rigor with human empathy. The model integrates plausible neurobiological pathways—hypothesizing that non-verbal semiotics may influence HPA-axis regulation and facilitate oxytocin release—with the goal of potentially mitigating the acute “cognitive blackout” that patients may experience during diagnostic procedures.

**Key findings:**

By structuring patient interaction, the CLEAR model could help patients regulate their acute stress response, preserving the patient's cognitive clarity and emotional resilience. Furthermore, by defining the radiologist's role within the multidisciplinary Breast Unit, the framework prevents the unproductive “chain of delegation” that frequently results in patient depersonalization.

**Conclusions:**

Re-establishing the radiologist's communicative role is vital for the integrity and efficiency of the Breast Unit. Implementing structured frameworks like the CLEAR protocol ensures diagnostic precision while safeguarding the patient's emotional energy for their subsequent oncological care.

## 1 The educational gap

Communication in diagnostic Breast Imaging represents today one of the deepest and least explored critical issues of the healthcare system. The original problem is educational and academic in its nature: formal training in physician-patient communication for radiologists remains historically limited (1). Historically, the radiologist was trained as an “image expert,” a health professional who prefers to be focused more on the screen than to the living body of the person. This formative gap emerges prominently during scientific conferences on diagnostic Breast imaging. It is almost paradoxical to observe how, in sessions dedicated to communication within breast radiology societies, only oncologists are invited to give lectures on communication and relationship with patients (2). Although the oncologists own great experience in the doctor-patient relationship, their model is structurally different: they manage different work rhythms and intervene when the disease is already a matter of fact. The radiologist, instead, operates in the time of uncertainties, much alike in “acute stress.” It is expert who first breaks the equilibrium of health. Radiology must therefore claim a communicative autonomy based on “ad hoc” rhythms: rapid, dense, and effective.

## 2 The two paths in the breast units: identity crisis and the risk of delegation

In the current era of Breast Units, the breast radiologist faces an identity crossroads. We have two ways to address the problem:

1. **The Path of Renouncement (The Impersonal Model):** The radiologist renounces this competence and undertakes a purely technical relationship. The burden of the word is shifted to the head of the Breast Unit (surgeon or oncologist), who, however, overloaded, tends to further delegate to the psychologist (3). But the psychologist has a supportive role; they cannot explain the technical nature of a biopsy-based suspicion with the authority of the one who identified it. This “chain of delegation” generates a psychological void around the patient, who feels treated like a bureaucratic file. A multidisciplinary process must not become an alibi for impersonality.
2. **Regaining Identity (The Modern-Traditional Model):** This means recovering the roles of the breast Radiologist, Surgeon, and Oncologist, each mastering their own communicative segment (4). In this scheme, the psychologist returns to their ideal function: they are not the one who gives the news, but the one who intervenes to deepen the emotional experience and the pending questions after the clinicians have spoken.

## 3 Clarity in the pathways

Today, patients are disoriented because all the professionals involved are generically called “senologists.” It is fundamental to provide clarity: the woman must know that the breast radiologist is the head of the diagnosis. The medical doctor, the interventional radiologist who performs the tests (ultrasound, MRI, CESM), carries out the biopsy and, above all, close the circle by receiving the histological examination (5). Contrary to a certain rhetoric, it is observed that the high volume of examinations is not a limit to the quality of the breast radiologist, but their guarantee. A radiologist is only as good as what they see (6, 7). Therefore, communication must be “tailored”: it must be able to fit into tight work rhythms without losing effectiveness, transforming every minute into a therapeutic act (8, 9).

## 4 The CLEAR model: a transcultural synthesis

From the collaboration between the breast radiologist and the psychiatrist with experience in European Union, Kurdish and Arab environments. The psychiatrist is an expert in dynamics between the direct rigor of East and Northern Europe and the relational mediation of the Arab world. This CLEAR protocol is proposed and here presented. This following model balances clinical truth with the necessary human empathy and can be adapted to the needs of the radiologist. The 5 Operational Phases are:

- **CONNECT:** The human link in the first 20 s. Looking at the woman in the eyes while wiping off the ultrasound gel means telling her: “I have seen your problem, and I am here with you; I know this isn't an easy moment for you.”
- **LISTEN:** Intercepting the “unspoken” (10). Before explaining, one must ask: “What worries you most at this moment?”. Listening allows for the modulation of the subsequent message, avoiding the cognitive blackout of the patient.
- **EXPLAIN:** The “stabilization through grounding” of the stress (11). Translating the suspicion into a clear operational plan: “We have found an area that requires further investigation; here is exactly what we will do.” Clarity reduces the anxiety of the unknown.
- **ASK:** Verification of the shock. The acute stress blocks memory. Asking the patient to repeat the steps of the pathway serves to bring her back to the operational reality.
- **REASSURE:** Total taking of responsibility (12, 13). It is not an “everything will be fine,” but an organizational reassurance: “I have already fixed the biopsy for Tuesday at 9:00 AM. I will follow you personally.” This certainty is the most powerful natural anxiolytic tool (Table 1 and Table 2).

We have to clarify that CLEAR is an acronym for a model which is clinically derived, practice-oriented conceptual framework. It was developed through an integrative synthesis of trauma-informed psychiatric practice, cross-cultural (culturally oriented) clinical experience, and established bad-news communication models, and refined through iterative clinical application. In Italy, the protocol is used in accredited training contexts, such as ECM (Continuing Medical Education) courses for psychologists, which attests to its practical usefulness and methodological reliability.

**Table 1:** CLEAR operational toolkit and implementation strategy.

Phase	Practical Tool (The “How”)	Time-Efficient Script (The “What”)	Potential Barrier
Connect	Eye contact/Physical proximity.	*"Good morning [Name], I am Dr. [Surname]."*	Cultural distance/Formalism.
Listen	The “20-second silence” rule.	*[Active silence during gel application]*	High-volume “rushed” mindset.
Explain	“Think-Aloud” technique.	*"I am scanning this area now to verify.."*	Technical jargon over-use.
Ask	The “Single Question” check.	*What is your main concern right now? Just to make sure I've been clear — what are the next steps as you understand them.*	Patient “cognitive blackout”.
Reassure	Operational “Next Steps”.	*"We will have the result by Tuesday."*	Uncertainty of results timeline.

**Table 2:** Comparative analysis of clinical communication protocols.

Comparative Dimension	SPIKES Protocol	SHARE Protocol	CLEAR Framework (Proposed)
Primary Clinical Context	Oncology, Palliative Care, & Inpatient Consultation	General Outpatient Consultations & Oncology	Diagnostic & Interventional Breast Radiology
Target Clinician	Oncologists, Palliative Care Specialists, Surgeons	Physicians, Nurses, Multi-disciplinary Teams	Breast Radiologists & Imaging Specialists
Setting & Timeframe	Seated, private, dedicated consultation room (20–45 min)	Standard outpatient medical consultation (15–30 min)	Compressed procedural & diagnostic windows (2–5 min)
Primary Objective	Structured disclosure of complex bad news and prognoses	Patient-centered communication & empathetic support	Acute psychological containment & procedural alignment
Methodological Strengths	Highly validated; clear step-by-step guidance for bad news	Strong emphasis on empathy, options, and reassurance	Reallocates routine procedural pauses (e.g., wiping gel)
Overlap with CLEAR	Focuses on checking comprehension and emotional support	Shares emphasis on warm, empathetic micro-interactions	Adapts core empathetic principles into high-speed workflows
Unique Radiological Advantages	Less adaptable to active physical examinations or rapid workflows	Requires dedicated conversational time outside the exam suite	Synchronizes verbal disclosure with manual scanning gestures without extending encounter time
Validation Status	Extensively validated empirically across multiple fields	Validated in oncology outpatient settings	Proposed conceptual model (Requires future empirical validation)

CLEAR is best understood not as a competing model, but as a clinically grounded adaptation shaped by everyday practice. Established frameworks such as SPIKES, NURSE, and SHARE have provided important, evidence-based structures for communicating difficult news in oncology. CLEAR does not seek to replace these contributions. Rather, it draws from them and extends their principles into the specific psychological and temporal realities of breast imaging, where disclosure often occurs within compressed timeframes and under acute emotional strain. By integrating trauma-focused stabilization with a culturally oriented approach and stepwise disclosure process adapted to the patient's individual context, its contribution lies in contextual application rather than theoretical novelty. Its clinical value should ultimately be examined through future empirical research.

The CLEAR model is designed as a “concurrent” rather than a “consecutive” framework: it does not require additional clinical time,but rather reallocates the existing technical windows—such as patient positioning and gel application—into high-impact communicative moments that prevent procedural delays and enhance patient cooperation.

## 5 Non-verbal language: the semiotics of gesture

In the ultrasound room, the physician's body communicates as much as their voice.

- **Prossemis:** Avoid the “technological wall.” Never turn your back to the patient while communicating. Moving frontally breaks the symbolic barrier.
- **The Medical Touch (When culturally and personally appropriate, and consistent with the patient's visible comfort with contact):** A soft touch on the shoulder during the explanation phase, ought to stimulate the release of oxytocin, anchoring the woman to the present (7).
- **The Tone of Voice:** A low and directive voice should act on the Vagus nerve, inducing a lowering of the heart rate and contrasting the cortisol storm (14).

## 6 Comparative hypothetical scenarios for didactic use

**Methodological Disclaimer:** The following clinical scenarios are constructed hypothetical vignettes created strictly for educational, illustrative, and conceptual purposes. They do not represent audited empirical data, clinical trial outcomes, or documented patient records, but they are designed to illustrate the practical application of the CLEAR framework in contrast to a conventional technical encounter.

### 6.1 Scenario 1: standard technical communication (conventional approach)

- **Clinical Context:** A 52-year-old patient undergoes a targeted breast ultrasound following a suspicious screening mammogram categorized as BI-RADS 4.
- **Radiologist's Interaction:** The clinician communicates the diagnostic finding while keeping their primary focus on the display monitor with minimal eye contact. The verbal exchange is brief and transactional: “*There is a suspicious finding that requires a biopsy. Please collect the procedural instructions at the front desk.”*
- **Observed Risk:** The absence of structured interpersonal anchoring may contribute to situational disorientation. The patient leaves the diagnostic suite in a state of heightened acute stress, potentially impairing her ability to process procedural instructions during the subsequent waiting period.
- **Commentary:** This transactional approach treats the patient primarily as a passive recipient of technical findings. The lack of non-verbal engagement misses an opportunity to offer immediate emotional containment, potentially leaving the patient's acute stress response unmanaged.

### 6.2 Scenario 2: integrated communication (applying the CLEAR framework)

- **Clinical Context:** The identical clinical scenario (BI-RADS 4 finding following a screening mammogram) within a high-anxiety diagnostic setting.
- **Radiologist's Interaction:** Utilizing existing technical pauses (such as wiping off the gel or completing image documentation), the radiologist pauses to establish direct eye contact (**CONNECT**), asks a brief open question to capture immediate concerns (**LISTEN**), outlines the clear diagnostic next steps (**EXPLAIN**), checks for procedural comprehension (**ASK**), and provides a concrete timeline for the biopsy (**REASSURE**), maintaining a calm, directive, and empathetic tone.
- **Intended Outcome:** The patient experiences a transition from acute cognitive blackout to structured clarity. While anxiety regarding the potential diagnosis remains, her sense of agency and comprehension is supported, facilitating compliance with the required diagnostic pathway.
- **Commentary:** By synchronizing routine clinical gestures with structured communication phases, the radiologist provides immediate cognitive and emotional grounding without extending the overall consultation timeframe. Rather than claiming a direct biological cure, this structured interaction is hypothesized to mitigate acute psychological disorientation and support patient coping.

## 7 The neurobiology of stress: why the word is a drug

Establishing an appropriate “interactional temperature” is increasingly recognized as a key component of patient-centered care, with supportive communication serving as a valuable clinical tool (15). Within this theoretical framework, the CLEAR protocol is conceptualized as an educational tool that is hypothesized to help manage the patient's stress response during acute diagnostic encounters.

In clinical settings, dismissive or overly detached communication styles are recognized in general psychological literature as potential psychosocial stressors. Such interactions are theorized to potentially engage threat-processing structures, such as the amygdala. It also may be consistent with models of hypothalamic-pituitary-adrenal (HPA) axis activation and transient cortisol elevation (16).

It is also believed that an empathetic and structured clinical approach—combining direct eye contact, a calm vocal tone, and clear operational guidance—is in accordance with literature suggesting that supportive micro-interactions might influence neuroendocrine regulation, including potential oxytocin-mediated stress attenuation (17).

Important Methodological Distinction: These biological mechanisms should be interpreted as theoretical frameworks and plausible neurobiological models derived from broader affective neuroscience, rather than direct, empirically proven cause-and-effect relationships within brief radiological examinations. There are currently no direct clinical trials confirming immediate hormonal or neurochemical changes resulting specifically from the radiologist's communicative gestures during an imaging session.

Rather than serving as a direct biological intervention, the structured communication proposed by the CLEAR protocol aims to provide cognitive containment and emotional grounding. By helping to frame the patient's initial psychological response during the diagnostic phase, the radiologist may assist in creating a more structured and manageable baseline for the patient's subsequent clinical journey.

## 8 Cultural adaptation

The radiologist must therefore be a “chameleon.” In Northern European cultures, the precision of the technical data wins. In Arabic and Mediterranean cultures, physical detachment is perceived as hidden bad news; here the warmth of the gesture is necessary to validate the medical truth.

Understanding these cultural barriers is vital for ensuring effective screening and diagnostic compliance Cultural adaptation within CLEAR extends beyond general sensitivity to differences in communication style. In several Middle Eastern contexts, for example, the term “cancer” is not always named directly. Instead, euphemistic expressions such as “that bad illness” are used, with the shared social understanding that the reference is to cancer. Such linguistic patterns reflect collective coping mechanisms but also reveal the existential weight carried by the diagnosis. When a patient perceives that they may be suffering from “that illness or bad illness, evil(malignant) illness- (Al-marad al-khabith),” the reaction is often not limited to medical concern; it may activate deeply rooted beliefs about fate, mortality, family responsibility, and spiritual meaning. At that moment, the individual's internal world—values, faith, identity, and future orientation—may feel profoundly destabilized. CLEAR integrates these cultural realities with neurobiological insights regarding stress-related cognitive narrowing. Acute threat perception can significantly impair comprehension and memory encoding, particularly when the diagnosis carries symbolic or existential meaning. In this sense, communication becomes a decisive clinical act. The manner in which the information is named, paced, and contextualized may determine whether the patient experiences the disclosure as disorganizing and overwhelming, or as structured and containable (16).

## 9 Situating CLEAR within the communication literature

While several established frameworks for clinical communication exist, such as the Calgary-Cambridge Guide or the SPIKES protocol, they are primarily designed for face-to-face consultations or the delivery of “breaking bad news” in a seated, conversational setting. However, diagnostic breast imaging presents a unique interaction: the clinician the patient and the imaging monitor.

The CLEAR model addresses a specific gap in current literature by integrating technical scanning performance with real-time patient engagement. Unlike broader models that focus on general history-taking, CLEAR is tailored for the high-anxiety environment of breast diagnostics where patients may enter a fight flight, or freeze response and where the physical act of the examination (ultrasound or mammography) often creates a communication barrier. By synchronizing the clinical gesture with structured verbal feedback, CLEAR ensures that the diagnostic process becomes a shared journey rather than a silent technical procedure. This situates CLEAR as a specialized, “point-of-care” communication tool that enhances scholarly rigor by addressing the intersection of manual dexterity, visual interpretation, and emotional support.

“For this paper, a literature search was conducted on PubMed using keywords such as “Breast cancer, Radiology Bad news”. We included peer-reviewed articles published between 2015 and 2025, focusing on practical scanning techniques and clinical guidelines.

While several established frameworks for clinical communication exist—most notably SPIKES (primarily developed for oncology) and SHARE (developed for general oncology outpatient settings)—these models are predominantly tailored for dedicated, seated, face-to-face consultations. Diagnostic and interventional breast imaging presents a structurally unique clinical interaction involving three concurrent focal points: the clinician, the patient, and the real-time imaging display.

The CLEAR framework does not seek to displace established, evidence-based tools like SPIKES or SHARE. Rather, it addresses a specific operational gap in current literature: the need for a “point-of-care” communication protocol synchronized with active technical imaging (ultrasound, mammography positioning, or biopsy execution).

To clarify its scholarly grounding, the key comparative dimensions among SPIKES, SHARE, and the proposed CLEAR framework are summarized below:

To integrate the table seamlessly into the text, you can add the following introductory and analytical paragraphs around it:

Rather than competing with traditional models, CLEAR operates as an adaptive adaptation shaped by the temporal and psychological realities of the radiology unit. While frameworks like SPIKES and SHARE excel in structured, seated conversations where diagnostic certainty has already been established, the breast radiologist must communicate during moments of acute diagnostic uncertainty.

As shown in Table 2, the distinct advantage proposed by CLEAR lies in its concurrent application: it does not demand extra consultation time, but instead converts mandatory procedural steps (such as transducer placement, gel removal, or image review) into high-impact communication anchors. This positions CLEAR as a specialized, practice-oriented framework designed to complement broader oncological communication guidelines.

## 10 Conclusion: communication is part of the care

If the radiologist abdicates their communicative role, the Breast Unit loses its initial pillar. Teaching communication to the radiologist means providing them with the tools to close the diagnostic circle with precision, avoiding the woman falling into despair and preserving her energies for the oncological therapy. Communication is not an “extra,” it is a fundamental medical act.

## Data Availability

The original contributions presented in the study are included in the article/Supplementary Material, further inquiries can be directed to the corresponding author.
