# Supplementary material for: The CLEAR model: a structured communication protocol for breast radiologists
Source: Front Health Serv. 2026 Aug 27;6:1890985. doi: 10.3389/frhs.2026.1890985 (PMC13558196; doi:10.3389/frhs.2026.1890985)
Supplement: Supplementary file 1 [file Datasheet1.pdf]

Table 1: CLEAR Operational Toolkit and Implementation Strategy

**Phase**	**Practical Tool (The "How")**	**Time-Efficient Script (The "What")**	**Potential Barrier**
**C**onnect	Eye contact / Physical proximity.	*"Good morning [Name], I am Dr. [Surname]."*	Cultural distance / Formalism.
**L**isten	The "20-second silence" rule.	*[Active silence during gel application]*	High-volume "rushed" mindset.
**E**xplain	"Think-Aloud" technique.	*"I am scanning this area now to verify..."*	Technical jargon over-use.
**A**sk	The "Single Question" check.	*"What is your main concern right now?"*	Patient "cognitive blackout".
**R**eassure	Operational "Next Steps".	*"We will have the result by Tuesday."*	Uncertainty of results timeline.

Table 2 The CLEAR Framework: A Neurobiological and Cross-Cultural Synthesis.

**Phase**	**Clinical Action**	**Neurobiological Driver (Stress)**	**Cultural Adaptation (Integration)**
**C**onnect	Eye contact & "Warm" welcome.	Triggers **Oxytocin**; inhibits initial Amygdala hijack.	Adjust eye contact/distance (e.g., more formal in Northern Europe, warmer in Mediterranean).
**L**isten	Active silence (15-30 sec).	Reduces **Cortisol** by lowering social threat perception.	Respect "high-context" communication styles (e.g., Middle Eastern patients may prioritize family narratives).
**E**xplain	Procedural "Normalization".	Prevents **"Cognitive Blackout"**; engages the Prefrontal Cortex.	Adapt technical language to health literacy levels across different backgrounds.
**A**sk	Confirmation of understanding.	Empowers the patient, reducing the sense of "learned helplessness."	Verify "saving face" (some cultures may say "yes" even if they haven't understood).
**R**eassure	Operational plan & certain dates.	Stabilizes the **HPA axis** for the long-term oncological journey.	Tailor the concept of "hope" (realistic vs. emotional) based on cultural spiritual frameworks.
